# Mortality from Copper Smelter Emissions: Pope Responds

**DOI:** 10.1289/ehp.10441R

**Published:** 2007-09

**Authors:** C. Arden Pope

**Affiliations:** Brigham Young University Provo, Utah E-mail: cap3@byu.edu

Grahame’s thoughtful and well-documented letter addresses one of the most important issues regarding our analysis of the mortality effects of a copper smelter strike in the U.S. Southwest (Pope et al. 2007). Grahame’s basic contentions are that changes in exposure to secondary sulfate alone were not sufficient to explain the observed mortality effects, and that the mortality effects were more likely due to changes in exposure to co-pollutants, such as biologically active metals and black smoke.

My coauthors and I agree with Grahame regarding several points. As he argues in his letter and as we briefly discussed in our article, the copper smelter strike also resulted in changes in exposure to metals and other co-pollutants. There is certainly evidence that metals, black carbon, and other by-products of incomplete combustion and high temperature industrial processes contribute to the pollution’s toxicity—as part of the complex mixture of fine particles.

We respectfully disagree in part with Grahame regarding the lack of evidence implicating secondary sulfate particles as contributing to adverse health effects. He asserts that the three “supporting” intervention studies “do not provide evidence that widespread secondary sulfate reductions were related to mortality reductions during the interventions.” However, in addition to changes in metals, black smoke, and other co-pollutants, one thing that all three intervention studies had in common was substantive changes in exposure to sulfate particles. In Utah Valley (Pope et al. 1992), the steel mill (largely from its coke ovens) was responsible for over 75% of the valley’s total sulfur oxide emissions. During winter-time temperature inversions, high concentrations of fine particulate matter with a relatively high proportion of sulfates occurred. The closure of the steel mill resulted in a disproportionately large drop in exposure to both metals, sulfates, and other mill-related pollutants.

Mortality reductions in Hong Kong were also associated with reductions in sulfur oxide exposure (Hedley et al. 2002). In Dublin, although sulfates were not measured, the banning of bituminous coal certainly resulted in an abrupt reduction in particulate pollution—including sulfate particles (Clancy et al. 2002).

In addition to the intervention studies discussed above, there is ample epidemiologic evidence that sulfate pollution, as part of complex mixtures, contributes to adverse health effects. For example, the Harvard Six-Cities Study (Dockery et al. 1993) and the American Cancer Society prospective cohort studies (Pope et al. 2002) of long-term air pollution exposure found both fine particulates and sulfate particles to be associated with mortality risk. A workshop of several research teams on source apportionment of particulate matter health effects found that the sulfate-related component of fine particles was most consistently associated with daily mortality (Thurston et al. 2005). The relative toxicity of sulfates per se and the additive or synergistic effects of related co-pollutants remains a matter of study and debate (Chen et al. 2006; Grahame and Schlesinger 2007). Nevertheless, epidemiologic studies of the adverse health effects of air pollution (Pope and Dockery 2006) have implicated fine particulate pollution from at least three general sources: coal combustion, high-temperature industrial processes, and traffic sources.

Overall, the literature suggests that sulfates—as part of mixtures of fine particles that include metals, black carbon, and other by-products of coal combustion, high-temperature industrial processes, and vehicle emissions—can contribute to adverse health effects. We reaffirm our conclusion that the results of our analysis of the mortality effects of the copper smelter strike “contribute to the growing body of evidence that ambient sulfate particulate matter and related air pollutants are adversely associated with human health.”
